# US Food and Drug Administration Action on Menthol Cigarettes and Flavored Cigars—A Pivotal Moment for Health Equity

**DOI:** 10.1001/jamanetworkopen.2022.17150

**Published:** 2022-06-01

**Authors:** Andrea C. Villanti, Kymberle Sterling, Shyanika W. Rose

**Affiliations:** Rutgers Center for Tobacco Studies, New Brunswick, New Jersey; Department of Health Behavior, Society and Policy, Rutgers School of Public Health, Piscataway, New Jersey; Department of Health Promotion and Behavioral Sciences, University of Texas School of Public Health, Dallas; Center for Health Equity Transformation, University of Kentucky, Lexington; Department of Behavioral Science, University of Kentucky College of Medicine, Lexington

In over a decade since the US Food and Drug Administration (FDA) gained regulatory authority over cigarettes, menthol cigarettes have eluded regulation. The most recent year of Federal Trade Commission cigarette sales data (2020) boasted the first increase in cigarette consumption in 20 years and the continued increase in menthol sales, comprising 37% of the cigarette market.^1^ In addition to changes in the market, new evidence has continued to underscore the potential public health benefit of banning menthol cigarettes, including rigorous studies like that of Leas et al,^2^ whose findings harness statistical methods designed to improve causal inference from observational studies. Like studies conducted in other cohorts, Leas et al^2^ provide strong evidence that menthol cigarettes are associated with smoking uptake, progression to regular use, and nicotine dependence in youth. The magnitude of these findings, their consistency across samples and over time, and their coherence with the underlying physiological effects of menthol support that removing menthol from cigarettes is likely to benefit public health, especially as it relates to reducing youth smoking initiation.

The modeling approaches used by Leas et al^2^ and others, including ourselves,^3^ focus on adjusting for potential confounders to control for underlying differences in the people who use menthol cigarettes compared with those who use nonmenthol cigarettes. This is valid and important, as we examine the association between menthol cigarettes and behavioral outcomes and produce conservative estimates of their role in smoking uptake and progression. However, in doing so, we lose the broader context of the people who use menthol cigarettes and the critical factors associated with menthol use.

Among past-month cigarette smokers in the US aged 12 years and older, 40% use menthol cigarettes, with higher prevalence in youth and young adults (aged 12–34 years; 48%–50%); Black individuals (85%), Asian and Asian American individuals (47%), those from multiracial backgrounds (51%), and Hispanic or Latinx individuals (50%); lesbian, gay, and bisexual individuals (46%–51%); those living in poverty (47%); and those who receive government assistance (46%).^4^ Greater use in these groups is no accident; it is the result of the predatory marketing of highly engineered menthol cigarettes to specific groups, with lower menthol levels designed to attract youth and young adults and higher menthol levels to attract long-term smokers. Targeted marketing of these products, particularly to young people, women, and Black populations, has occurred, in part, through print advertisement placements in magazines with Black and younger readership, price discounts for menthol cigarettes in direct mail, point-of-sale marketing in Black and lower-income communities, and lower prices for menthol cigarettes near public schools.^5^ These marketing efforts have been augmented by decades of tobacco company investment in Black-led community-based and national organizations, sponsorships for community activities, support for civil rights organizations, hiring of Black workers, and, recently, public disavowal of systemic racism.^6,7^ These activities stand in direct contrast to the business imperatives of the cigarette companies and have not resulted in any change to their marketing practices, which have grown the presence of menthol cigarettes in Black, Hispanic, and lower-income communities.

Notably, menthol cigarettes and flavored filtered cigars and cigarillos share a similar history of targeted marketing and patterns of use. These products are inexpensive, mass-merchandised products often marketed at retail outlets in neighborhoods with large numbers of Black residents, youth, and young adults. They are also heavily advertised online and on social media platforms. Like menthol cigarette brands, flavored filtered cigar and cigarillo brands have appropriated aspects of Black and urban culture, including hip-hop music, to normalize and promote brand loyalty and product use. Their users are more likely to be young adults (aged 18–24 years vs ≥25 years), Black and Hispanic, and daily menthol cigarette users.^8^ In addition, young people who start with a menthol cigarette or flavored cigar are more likely to continue smoking a year later.^3^ The availability of menthol cigarettes and flavored cigars threatens to exacerbate disproportionate rates of tobacco-caused morbidity and mortality among Black and other vulnerable populations.

Researchers and public health advocates have highlighted a menthol cigarette ban as a remedy for social injustice^4,7^ and an opportunity to increase health equity, particularly for Black populations in the US.^9^ As described in the popular press and the scientific literature, a significant impediment to the passage of state and local policies restricting menthol cigarettes has been tobacco company recruitment of Black lobbyists and consultants to argue against such policies.^7,10^ Tobacco company–tied claims have exploited legitimate concerns about racist policing and advocated against the passage of local and state menthol bans, spreading concerns and misinformation about an illicit market and increased crime and policing in Black communities stemming from menthol and flavor bans.^7,10^ With 37% market share and 40% menthol cigarette smoking prevalence, there is no question that tobacco companies will fight an FDA menthol cigarette and flavored cigar ban through court battles, advocacy, the perpetuation of misinformation and disinformation, and all other means at their disposal. However, further delay will have tremendous costs to public health, including millions of extra smokers, millions of life-years lost, and hundreds of thousands of premature deaths, with disproportionate impacts on Black individuals.^7^

As of 2018, only 6.3% of the US population was covered by a flavored tobacco policy at the state or local level, and only 0.9% were covered by a more robust policy that included restrictions on the sale of menthol cigarettes.^9^ Youth, African American, Hispanic, and American Indian/Alaska Native populations, along with populations of lower education, were less likely to be covered by these stronger flavored tobacco policies.^9^ FDA’s anticipated product standards on menthol cigarettes and flavored cigars implemented at the national level will rapidly expand coverage of stronger flavored tobacco policies to 100% of the population. Importantly, these policies are likely to improve equity in coverage and health benefits for populations at greater risk of menthol cigarette and flavored cigar use, including young people. As such, the potential equity impacts of menthol cigarette and flavored cigar bans are due to the very characteristics of those most burdened with the use of these products, not the population made homogeneous in our statistical comparisons.

Maximizing the public health benefit of FDA’s policies on menthol cigarettes and flavored cigars will require a range of activities: (1) developing culturally appropriate communication efforts to raise awareness and counter misperceptions and disinformation about the goals of flavored tobacco policies; (2) ensuring that policy enactment and enforcement focus on retailers and manufacturers as proposed; (3) limiting policy exemptions that may lead to loopholes and inequitable implementation; and (4) scaling up culturally appropriate, free, and accessible resources to support tobacco cessation. FDA’s proposed bans on menthol cigarettes and flavored cigars offer a watershed moment to refocus our efforts and provide tobacco prevention and cessation resources for the people and communities who have been targeted by tobacco marketing and, by design, are at the greatest risk of tobacco use and its harms.
